# Nutritional Intervention With Perennial Ryegrass Modulates UA Metabolism in Goslings via Gut Microbiota–Antioxidant Pathway Modulation

**DOI:** 10.1002/fsn3.71760

**Published:** 2026-04-17

**Authors:** Muhammad Arslan Asif, Zeshan Zulfiqar, Bahar E. Mustafa, Saira Saif, Zhichang Wang, Yalei Cui, Sun Hao, Liu Boshuai, Yinghua Shi

**Affiliations:** ^1^ Department of Animal Nutrition and Feed Science, College of Animal Science and Technology Henan Agricultural University Zhengzhou Henan China; ^2^ Melbourne Veterinary School, Faculty of Science The University of Melbourne Melbourne Victoria Australia; ^3^ Henan Key Laboratory of Innovation and Utilization of Grassland Resources Zhengzhou China; ^4^ Henan Forage Engineering Technology Research Center Zhengzhou Henan China

**Keywords:** antioxidant metabolites, gut microbiota, hyperuricemia, ROS, uric acid

## Abstract

Hyperuricemia (HUA) is a metabolic disorder characterized by excessive uric acid (UA) accumulation resulting from increased hepatic production and impaired renal and intestinal excretion. Nutritional interventions using plant‐based feed resources rich in bioactive compounds have shown promise in regulating urate metabolism in poultry. Perennial ryegrass, a forage abundant with dietary fiber and polyphenols, exhibits antioxidant and anti‐inflammatory properties that may modulate UA metabolism. In the present study, Wanpu goslings were used to investigate the protective effects of perennial ryegrass against HUA and UA‐induced oxidative stress. In the present study, Wanpu goslings were assigned to three dietary treatments: (i) concentrate‐only diet (CD), (ii) concentrate diet supplemented with 30% perennial ryegrass (70% concentrate:30% ryegrass; GD1), and (iii) concentrate diet supplemented with 50% perennial ryegrass (50% concentrate:50% ryegrass; GD2). While CD and GD1 supported higher average daily gain compared to GD2. Inclusion of perennial ryegrass significantly reduced serum UA and creatinine (Cr) levels. Noticeably, ryegrass supplementation decreased hepatic xanthine oxidase (XOD) activity and enhanced the expression of urate transporters (ABCG2, OAT1, and OAT3) in intestinal and renal tissues, indicating improved urate excretion. Moreover, perennial ryegrass improved intestinal morphology and barrier integrity, attenuated oxidative stress through activation of the Nrf2‐ABCG2 signaling pathway, increased antioxidant enzyme activities, and reduced pro‐inflammatory cytokine levels. Additionally, 16S rRNA sequencing revealed enrichment of beneficial microbial genera, including *Alistipes*, *Ruminococcus*, and *Akkermansia*, in ryegrass‐fed goslings. Metabolomic profiling further demonstrated decreased concentrations of HUA‐associated metabolites xanthine, hypoxanthine, and inosine, and elevated levels of bioactive phenolic compounds such as caffeic acid, kaempferol, quercetin, and ferulic acid. Network analysis suggested antagonistic interactions between polyphenolic metabolites and urate‐promoting intermediates. These findings indicate that moderate inclusion of perennial ryegrass (30%) in concentrate‐based diets may serve as a sustainable nutritional strategy to modulate UA metabolism, antioxidant capacity, and gut microbial composition in intensive goose production systems.

## Graphical abstract

Perennial ryegrass supplementation modulates UA‐metabolism in goslings by reducing serum uric acid and creatinine through inhibition of hepatic xanthine oxidase and upregulation of urate transporters (ABCG2, OAT1, OAT3). It enhances antioxidant capacity via Nrf2 activation, improves intestinal barrier integrity, and enriches beneficial gut microbiota (*Alistipes*, *Ruminococcus*, *Akkermansia*). Metabolomic changes include decreased purine metabolites and increased polyphenols, supporting improved urate metabolism and overall health.

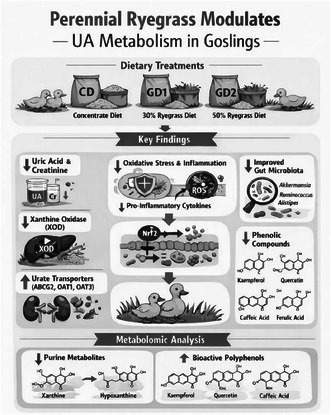

## 1 Introduction

Hyperuricemia is a metabolic and inflammatory disorder characterized by excessive accumulation of UA in the body, affecting both humans and avian species (Shao et al. 2017; Xi et al. 2019). Persistent elevation of UA promotes the formation and deposition of monosodium urate crystals in joints and soft tissues, triggering inflammatory responses and tissue injury (Desai et al. 2017). Epidemiological evidence over the past decade indicates HUA prevalence across China, ranging from 5.46% to 19.30%, specifically 9.2%–26.2% and 0.7%–10.5% in males and females, respectively (Multidisciplinary Expert Task Force on Hyperuricemia and Related Diseases 2017). In China, rising hyperuricemia prevalence poses a growing health concern and is strongly associated with gout, renal dysfunction, diabetes, and cardiovascular disease (He et al. 2022). Uric acid, as the final product of purine metabolism, is produced through the regulation of xanthine oxidase (XOD) and xanthine dehydrogenase (XDH). While both catalyze similar reactions, XDH uses NAD^+^ as an electron acceptor, whereas XOD utilizes O_2_, resulting in the production of reactive oxygen species such as superoxide anions (Veljković et al. 2020). Physiologically, approximately 30% of uric acid is excreted through the intestine, largely mediated by ABCG2, whereas about 70% is handled by the kidneys through coordinated secretion and reabsorption processes involving ABCG2, OAT1, OAT3, URAT1, and GLUT9. The expression and activity of these transporters are dynamically regulated under different environmental and pathological conditions (Xu et al. 2017).

Unlike most mammals such as mice, which possess uricase to further degrade UA into a more soluble form, humans and poultry lack functional uricase. This evolutionary deficiency predisposes them to UA accumulation and HUA (Guo et al. 2005; Xi et al. 2022). In China, geese production is extensive, with an annual consumption of approximately 500–600 million birds. However, gout and hyperuricemia are highly prevalent during the growth phase, affecting a major number of geese and resulting in considerable economic losses (An et al. 2020; Wang et al. 2021). Goslings are more susceptible to HUA and gout within the first 1–20 days post‐hatch (Guo et al. 2005; Zhang et al. 2018; An et al. 2020; Xi et al. 2020), as reported in a Muscovy duck farm in Henan Province and Shandong Province (Wei et al. 2020; Chen et al. 2021). Dietary factors, notably high‐protein and high‐fat feeds rich in purines from animal viscera, fishmeal, and nucleoproteins, are strongly associated with diet‐induced HUA, whereas low fiber intake further increases the risk (Wang et al. 2011; Xi et al. 2019; Dehlin et al. 2020).

Emerging evidence suggests that gut microbiota dysbiosis plays a pivotal role in metabolic and inflammatory disorders, including gout and kidney diseases (Chang et al. 2019; Zhou et al. 2023). Disruption of intestinal tight junction proteins (TJPs) compromises barrier integrity, leading to increased gut permeability and translocation of pathogenic bacteria (Williams et al. 2015). This process activates NF‐κB signaling and promotes the release of pro‐inflammatory cytokines such as interleukin‐1β (IL‐1β), interleukin‐18 (IL‐18), and tumor necrosis factor‐α (TNF‐α), thereby amplifying ROS‐mediated systemic inflammation (Xi et al. 2019; Yang et al. 2024). Sustained inflammation impairs nutrient absorption, disturbs fluid balance, and contributes to renal dysfunction in poultry (Awad et al. 2017).

Geese possess a strong ability to digest fibrous plant materials, including grasses and leafy vegetables (Zhang et al. 2013). Forage supplementation has been shown to enrich cellulose‐degrading bacterial families such as *Bacteroidaceae* and *Lachnospiraceae* while reducing opportunistic pathogens including *Enterococcus* and *Escherichia‐Shigella* (Xu et al. 2017; Chen et al. 2018). Perennial ryegrass, widely cultivated in China, is a nutrient‐dense forage containing bioactive compounds such as flavonoids, polyphenols, and glycosides with demonstrated antioxidant and anti‐inflammatory properties (Chilibroste et al. 2000). These polyphenolic constituents mitigate oxidative stress by scavenging reactive oxygen species (ROS), inhibiting uric acid‐producing enzymes including XOD and XDH, and enhancing endogenous antioxidant enzymes (Gessner et al. 2017). Such mechanisms collectively strengthen hepatic and renal antioxidant capacity in livestock (López‐Andrés et al. 2014; Yahfoufi et al. 2018). Importantly, plant‐derived antioxidants can activate nuclear factor erythroid 2‐related factor 2 (Nrf2), a key regulator of cellular redox balance (Ali et al. 2022). Upon activation, Nrf2 dissociates from Keap1 and translocates to the nucleus, where it binds antioxidant response elements (AREs) within the promoter regions of target genes, including the urate efflux transporter ABCG2 (Singh et al. 2010). Upregulation of ABCG2 enhances intestinal and renal uric acid excretion, thereby supporting urate homeostasis while attenuating ROS‐mediated tissue injury. Goslings with high susceptibility to UA accumulation, activation of the Nrf2‐ABCG2 axis by perennial ryegrass may represent a nutritional strategy to simultaneously enhance urate clearance, modulate gut microbiota composition, and reduce systemic inflammation. Perennial ryegrass bioactive compounds have been shown to beneficially modulate intestinal microflora of Beijing‐you chickens and exhibit therapeutic effects in animal disease models (Choi et al. 2017; Zheng et al. 2021).

In the present study, we hypothesized that the fiber‐rich and polyphenol‐enriched composition of perennial ryegrass may beneficially influence gut microbial composition, uric acid metabolism, and antioxidant status in Wanpu goslings, potentially through activation of the Nrf2‐ABCG2 signaling pathway. By simultaneously modulating microbial homeostasis and UA excretion, perennial ryegrass supplementation may represent a cost‐effective nutritional approach to support urate balance and oxidative resilience in intensive goose production systems.

## 2 Materials and Methods

All procedures involving animals were approved by the Research Bioethics Committee of Henan Agricultural University (HENAU‐202305) and the Animal Ethics Committee of the College of Animal Science. The experiment was conducted in accordance with institutional guidelines and standards.

### 2.1 Animals and Housing

A total of 450 one‐day‐old Wanpu goslings of mixed sex were obtained from Henan Daidai Goose Agriculture and Animal Husbandry Development Co. Ltd. (Zhumadian, China). Birds with comparable initial body weights were randomly assigned to three dietary treatments, each comprising six replicates of 25 goslings. Baseline serum UA was not measured prior to treatment to minimize stress and blood sampling in newly hatched birds. Therefore, randomization and comparable initial body weight were used to reduce baseline bias. The experiment was conducted over 21 days in a semi‐controlled housing facility equipped with electric heating and plastic mesh flooring. Fiberglass feeders and automatic bell drinkers were provided to ensure ad libitum access to feed and water. Lighting was maintained at an intensity of 20 lx for 24 h during the first 3 days, followed by an 18‐h light and 6‐h dark cycle thereafter. The ambient temperature was initially set at 35°C for the first 3 days and subsequently reduced by 3°C per week until Day 21. Mortality was recorded daily for each replicate and summarized weekly as the number of dead birds per replicate. Mortality data were analyzed using one‐way ANOVA based on replicate‐level values. Body weight and feed consumption were measured on Days 7, 14, and 21 to determine growth performance parameters, including average daily gain (ADG), average daily feed intake (ADFI), and feed conversion ratio (FCR). These parameters were calculated as:

$$\begin{array}{c} \text{ADFI}\;\left(g/\text{bird}/\mathrm{day}\right)=\text{Total feed consumed during the period}\\ \;÷\left(\text{Number of birds}\times \text{Number of days}\right) \end{array}$$

$$\begin{array}{c} \mathrm{ADG}\;\left(g/\text{bird}/\mathrm{day}\right)=\left(\text{Final body weight}-\text{Initial body weight}\right)\\ \;÷\text{Number of days} \end{array}$$

FCR=ADFI÷ADG

### 2.2 Experimental Design and Treatments

The Goslings with an average initial body weight of 90 ± 5 g were randomly allocated into three experimental groups: a concentrate diet group (CD, *n* = 150) and two ryegrass‐supplemented groups (GD1, *n* = 150; GD2, *n* = 150). Birds in the CD group received a concentrate diet formulated according to the manufacturer's recommendations (Table S1). In the supplemented groups, concentrate feed was combined with chopped perennial ryegrass at ratios of 70% concentrate:30% ryegrass for GD1 and 50% concentrate:50% ryegrass for GD2. During the first 7 days, goslings had ad libitum access to feed. Feed refusals were collected and weighed the following morning to determine daily feed intake. From Day 8 to Day 21, feeding was restricted and provided three times daily at 8‐h intervals. Remaining feed was collected and weighed 2 h after each feeding session to calculate feed consumption. The nutrient composition of perennial ryegrass is presented in (Table S2). The nutrient profiles of the experimental diets analyzed are summarized in Table 1.

**TABLE 1:** Nutritional composition of diets.

Feed components	CD	GD1	GD2
Crude protein (%)	19.57	18.98	18.5
Metabolizable energy (MJ/kg)	11.4	10.83	10.45
Crude fiber (%)	4.5	11.1	15.5
Neutral detergent fiber (%)	11.7	19	23.86
Acid detergent fiber (%)	5.56	10.5	13.77
Ash (%)	12.89	7	7.52
Calcium (%)	0.85135	0.87	0.876
Total phosphorus (%)	0.36005	0.39	0.41

*Note:* CD = Concentrate diet:Perennial ryegrass (100:0), GD1 = Concentrate diet:Perennial ryegrass (70:30), GD2 = Concentrate diet:Perennial ryegrass (50:50).

### 2.3 Sample Collections and Preparations

On Days 7, 14, and 21, three goslings with body weights representative of the group mean were randomly selected from each replicate following a 12‐h fasting period. Blood samples were collected via the cutaneous ulnar vein and centrifuged at 3000×*g* for 10 min at 4°C to obtain serum, which was aliquoted and stored at −20°C until further analysis. After blood collection, the selected birds were humanely euthanized by cervical dislocation. Cecal digesta samples were aseptically collected into sterile 5 mL centrifuge tubes and immediately stored at −80°C for subsequent analyses. Tissue samples from the ileum, cecum, liver, and kidney were excised, labeled, and preserved at −80°C. Additional portions of cecum, liver, and kidney tissues were fixed in 4% and 10% neutral buffered formalin for histological examination.

### 2.4 Analysis of HUA Biochemical Parameters, Enzymes and Transporters

Serum biochemical parameters related to HUA, including UA, Cr, blood urea nitrogen (BUN), and lipid metabolism indices, were measured using commercial enzymatic assay kits (Nanjing Jiancheng Bioengineering Institute, Nanjing, China) according to the manufacturer's protocols. Serum activities of UA‐producing enzymes XOD and XDH were also determined using corresponding assay kits. The concentrations of urate transporters, including ATP‐binding cassette subfamily G member 2 (ABCG2), organic anion transporter 1 (OAT1), organic anion transporter 3 (OAT3), glucose transporter 9 (GLUT9), and urate transporter 1 (URAT1), were quantified using enzyme‐linked immunosorbent assay kits (Shanghai Meilian Biology Technology, Shanghai, China).

### 2.5 Measurement of the Serum Levels of Inflammatory, Anti‐Inflammatory Cytokines, Oxidant and Antioxidant Enzymes

Serum and cecal samples were collected into sterile, enzyme‐free centrifuge tubes for biochemical analyses. Concentrations of pro‐inflammatory and anti‐inflammatory cytokines were quantified using commercially available ELISA kits (Jiangsu Meimian Industrial Co. Ltd.) following the manufacturer's instructions. Oxidative stress markers and antioxidant enzyme activities in serum were also determined using ELISA kits (Shanghai Meilian Biology Technology, Shanghai, China). All measurements were performed in triplicate to ensure analytical accuracy and reproducibility.

### 2.6 Hematoxylin and Eosin Staining of Ileum

Initially, Ileal samples were fixed in 4% paraformaldehyde for 24 h at room temperature to preserve tissue architecture. After fixation, tissues were gradually dehydrated using increasing concentrations of ethanol, cleared in xylene, and embedded in paraffin. The paraffin‐embedded blocks were sectioned at 7 μm thickness with a Leica RM2235 microtome and mounted onto glass slides for staining. For histological evaluation, sections were first deparaffinized in xylene and rehydrated through a graded ethanol series to distilled water. The slides were stained with Harris hematoxylin to visualize nuclei, differentiated using an acetic acid–ethanol solution, and subsequently treated with saturated lithium carbonate to enhance nuclear contrast. Sections were then counterstained with eosin Y to highlight cytoplasmic structures. After dehydration and clearing, the slides were coverslipped for microscopic examination. Images were acquired using a Nikon microscope equipped with NIS‐Elements software. Intestinal morphology, including villus height, villus width, and crypt depth, was measured using Image‐Pro Plus 6.0 software (Media Cybernetics, Rockville, MD, USA).

### 2.7 Periodic Acid‐Schiff (PAS) Staining Cecal Tissues

Cecal sections stained with periodic acid–Schiff (PAS) were observed under a light microscope at 40× magnification to assess mucosal architecture. Goblet cells were identified based on PAS‐positive staining and quantified using ImageJ 6.0 software (MD, USA). Histological evaluations were performed according to established morphological criteria.

### 2.8 Measurement of Gut Permeability

Intestinal barrier integrity was evaluated by assessing tight junction protein expression in cecal tissues. ZO‐1 and Claudin were analyzed by immunofluorescence staining, and fluorescence intensity was quantified using ImageJ 6.0 software (MD, USA). The mRNA expression levels of ZO‐1, Claudin, mucin‐2 (MUC‐2), and its regulatory genes Notch1 and Notch2 were determined to further characterize barrier function. Serum diamine oxidase (DAO) concentration, a marker of intestinal permeability, was measured using a commercial ELISA kit (Shanghai Mlbio Biotechnology Co. Ltd.) according to the manufacturer's protocol.

### 2.9 Immunofluorescence Analysis of Nrf2 and ABCG2

Immunofluorescence (IF) analysis of kidney tissues was performed by using the method described by (Zulfiqar et al. 2025). Immunofluorescence intensities for 3D surface plots were analyzed using ImageJ software analysis.

### 2.10 RNA Extraction and Quantitative Reverse Transcription Polymerase Chain Reaction (RT‐qPCR) and Western Blot Analysis

Gene expression analysis was performed by quantitative PCR following previously described procedures (Ali et al. 2024). Gene‐specific primers were designed using Primer3web (version 4.1.0) and are listed in (Table S3). Protein expression in cecal, renal, and hepatic tissues was evaluated by Western blot analysis according to established protocols (Ali et al. 2022). Details of the primary antibodies used and their corresponding molecular weights (kDa) are provided in (Table S4).

### 2.11 Analysis of 16S rRNA Gene Sequencing

Total microbial genomic DNA was extracted from cecal chyme samples using the E.Z.N.A. Soil DNA Kit (Omega Bio‐tek, Norcross, GA, USA) following the manufacturer's protocol. DNA integrity and concentration were verified by 1% agarose gel electrophoresis and NanoDrop 2000 spectrophotometry (Thermo Scientific, USA), and samples were stored at −80°C until further processing. The V3–V4 hypervariable region of the bacterial 16S rRNA gene was amplified using primers 338F and 806R in a T100 thermal cycler (Bio‐Rad, USA). PCR products were purified using a PCR Clean‐Up Kit (YuHua, Shanghai, China), quantified with a Qubit 4.0 fluorometer (Thermo Fisher Scientific, USA), and sequenced on the Illumina PE300/PE250 platform (Illumina, San Diego, USA) by Majorbio Bio‐Pharm Technology Co. Ltd. (Shanghai, China). Sequence data were processed using QIIME2 (version 2020.2). Amplicon sequence variants (ASVs) were assigned taxonomy using a Naïve Bayes classifier against the SILVA 16S rRNA reference database (v138). Functional potential of the microbial communities was predicted using PICRUSt2 based on ASV representative sequences. Downstream bioinformatics and statistical analyses, including alpha and beta diversity, linear discriminant analysis effect size (LEfSe), redundancy analysis (RDA), and Spearman correlation analysis, were conducted via the Majorbio Cloud platform (https://cloud.majorbio.com) using standard pipelines (Mothur, Vegan package, and related tools).

### 2.12 Serum Metabolomics Analysis

Serum metabolomic profiling was performed using a Thermo UHPLC‐Q Exactive HF‐X system coupled with an ACQUITY HSS T3 column (100 mm × 2.1 mm, 1.8 μm; Waters, USA) at Majorbio Bio‐Pharm Technology Co. Ltd. (Shanghai, China). For metabolite extraction, 100 μL of serum was mixed with 400 μL of extraction solvent (acetonitrile: methanol, 1:1, v/v) containing 0.02 mg/mL L‐2‐chlorophenylalanine as an internal standard. After centrifugation, the supernatant was collected for LC–MS/MS analysis. Quality control (QC) samples were prepared and analyzed under identical conditions.

Mass spectrometric detection was conducted using an electrospray ionization (ESI) source operating in both positive and negative ion modes. Key parameters included a capillary temperature of 325°C, auxiliary gas heater temperature of 425°C, sheath gas flow rate of 50 psi, auxiliary gas flow rate of 13 psi, and spray voltage of ±3500 V. Data were acquired in data‐dependent acquisition (DDA) mode over a mass range of 70–1050 m/z, with resolutions of 60,000 for full MS and 7500 for MS/MS. Raw data were processed using Progenesis QI software (Waters, Milford, USA) for baseline correction, peak detection, alignment, and normalization. Metabolites were annotated by comparison with the HMDB, METLIN, and the Majorbio in‐house database. Multivariate analyses, including principal component analysis (PCA) and orthogonal partial least squares discriminant analysis (OPLS‐DA), were performed using the R package “ropls.” Differential metabolites were identified based on variable importance in projection (VIP > 1) and Student's *t*‐test (*p* < 0.05). Functional annotation and pathway enrichment analyses were conducted using the KEGG database, and statistical enrichment was performed using the Python package “scipy.stats.”

### 2.13 Statistical Analysis and Data Visualization

Data are presented as mean ± SD. Statistical analyses were conducted using SPSS version 20.0 (SPSS Inc., Chicago, IL, USA). Normality was first assessed using the Shapiro–Wilk test. Three groups' growth performance data were analyzed by one‐way analysis of variance (ANOVA), followed by the least significant difference (LSD) post hoc test. Comparisons between two groups were performed using an unpaired two‐tailed Student's *t*‐test. Statistical significance was set at *p* < 0.05. Spearman correlation analysis based on Euclidean distance was conducted using GraphPad Prism version 8.3.0 and Origin 2021. Pearson correlation analysis to evaluate associations among host biomarkers was performed using the OECloud platform (https://cloud.oebiotech.cn).

## 3 Results

### 3.1 Effects of Perennial Ryegrass on Growth Performance and Serum HUA Biomarkers and Serum Lipid Metabolic Profile

**FIGURE 1:**
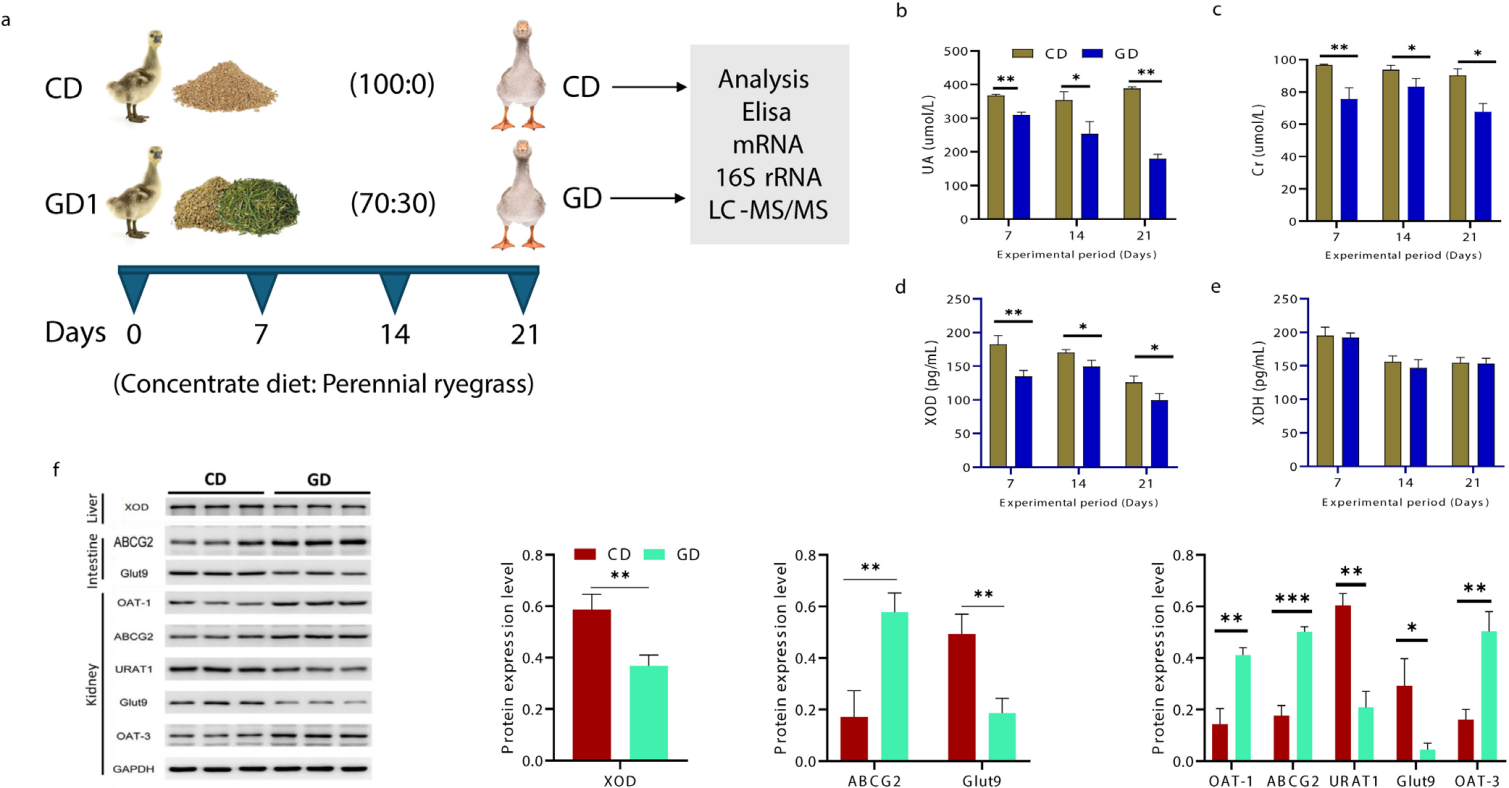
Effects of perennial ryegrass supplementation on growth performance, HUA biomarkers, and UA metabolism. (a) Experimental layout. (b, c) Serum levels of uric acid (UA) and creatinine (Cr), respectively. (d, e) Serum concentrations of xanthine oxidase (XOD) and xanthine dehydrogenase (XDH). (f) Western blot analysis of UA producing enzymes and transporters in the liver (XOD), intestine (ABCG2, GLUT9), and kidney (OAT1, ABCG2, URAT1, GLUT9, OAT3). GAPDH was used as internal control. Data are expressed as mean ± SD. The asterisks symbol indicates significant differences: *p < 0.05, **p < 0.01, ***P < 0.001.

The effects of experimental treatments on growth performance are presented in Table 2. There were no significant differences in ADFI among experimental groups. The CD and GD1 groups have higher ADG (*p* < 0.05) than GD2, as well as FCR being higher in the GD2 group. The ADG of the CD group was numerically higher than GD2 but statistically (*p* > 0.05) not significant. The mortality rate was significantly higher (*p* < 0.05) in the CD group than in the GD1 and GD2 groups. Lower ADG and higher FCR in the GD2 group were due to a lower crude protein ratio in the diet, and GD2 is not recommended for commercial rearing and was therefore excluded from further analysis (Figure 1a).

**TABLE 2:** Effect of different diets on production performance of goslings.

Parameters	CD	GD1	GD2	*p*
Week 1				
ADFI (g)	80.38 ± 0.91	81.42 ± 0.71	80.67 ± 0.21	0.137
ADG (g)	49.29 ± 0.62^a^	48.5 ± 0.81^a^	45 ± 1.3^b^	0.002
FCR	1.63 ± 0.04^b^	1.68 ± 0.05^b^	1.78 ± 0.04^a^	0.01
Mortality	0.67 ± 0.81	0.33 ± 0.51	0.33 ± 0.51	0.58
Week 2				
ADFI (g)	131.5 ± 2.38	132.66 ± 3.51	134.9 ± 2.61	0.357
ADG (g)	51.8 ± 1.36^a^	47.73 ± 1.16^b^	36.5 ± 1.17^c^	0.003
FCR	2.54 ± 0.11^b^	2.78 ± 0.13^b^	3.69 ± 0.13^a^	0.001
Mortality	1 ± 0.63	0.33 ± 0.51	0.33 ± 0.51	0.089
Week 3				
ADFI (g)	175.29 ± 2.58	177.190 ± 3.29	178 ± 2.65	0.527
ADG (g)	105.6 ± 2.76^a^	101.8 ± 4.1^a^	80.1 ± 4.32^b^	0.002
FCR	1.66 ± 0.03^b^	1.74 ± 0.1^b^	2.20 ± 0.1^a^	0.001
Mortality	1 ± 0.89^a^	0.33 ± 0.51^ab^	0^b^	0.032

*Note:* CD = Concentrate diet:Perennial ryegrass (100:0), GD1 = Concentrate diet:Perennial ryegrass (70:30), GD2 = Concentrate diet:Perennial ryegrass (50:50). ^a,b,c^Means within row different superscripts differ significantly (*p* < 0.05). Mortality was recorded as the number of dead birds per replicate.

Hyperuricemia is characterized by high serum UA level. Serum UA and Cr levels reduced gradually in the ryegrass‐fed group at Days 7, 14 and 21 (Figure 1b,c). In contrast, BUN levels did not differ significantly among treatments (Figure S1a). However, dietary inclusion of perennial ryegrass significantly modulated serum UA and Cr concentrations. Additionally, the GD groups exhibited improved lipid profiles, with significantly lower serum total cholesterol (T‐CHOL), triglycerides (TG), and low‐density lipoprotein (LDL) levels relative to the CD group (Figure S1b–d). The serum high‐density lipoprotein (HDL) was observed at higher levels in the GD group (Figure S1d). These results suggest that perennial ryegrass inclusion may influence serum UA levels and improve lipid metabolism in Wanpu geese.

### 3.2 The Effects of Dietary Perennial Ryegrass on UA Producing Enzymes

Key enzymes involved in uric acid production are XOD and XDH. The concentrate‐fed group showed increased levels of XOD in blood (*p* < 0.05) and their concentration was decreased in the GD group, while XDH showed non‐significant (Figure 1d,e). The transcriptional abundance (XOD and XDH) and protein levels of XOD were analyzed in the liver. High transcriptional abundance was observed in the CD group, while they had decreased expression in the GD group (Figure S2f). A similar trend was observed for XOD at the protein level (Figure 1f). These results indicate that perennial ryegrass supplementation may modulate the uric acid metabolism in goslings.

### 3.3 The Effects of Dietary Perennial Ryegrass on Intestinal and Renal UA Transporters

Uric acid secretion is primarily facilitated by the kidneys (70%) and the intestine (30%) through coordinated activity of urate transporters such as ABCG2, OAT1, OAT3, URAT1, and GLUT9. Serum ELISA detected measurable levels of ABCG2, OAT1, and OAT3, with significantly higher relative concentrations (*p* < 0.05) in the GD group compared with the CD group (Figure S2a–c). In contrast, serum levels of the reabsorptive transporters URAT1 and GLUT9 were lower in the GD groups (Figure S2d,e). The transcriptional abundance levels of transporters including ABCG2, OAT1, and OAT3 were higher in the GD group (both in ileum and kidney) relative to CD (Figure S2g,h). Protein expression levels for these transporters in ileum and kidney were also consistent with the ELISA and RT‐qPCR results (Figure 1f). As these proteins are membrane‐associated transporters primarily localized in intestinal and renal epithelial tissues, circulating levels may reflect protein fragments or cellular turnover rather than direct functional transporter abundance. Therefore, conclusions regarding transporter modulation are primarily supported by tissue‐level mRNA and protein expressions.

### 3.4 Perennial Ryegrass Improved Intestinal Morphology and Intestinal Integrity by Modulating Tight Junction Proteins

**FIGURE 2:**
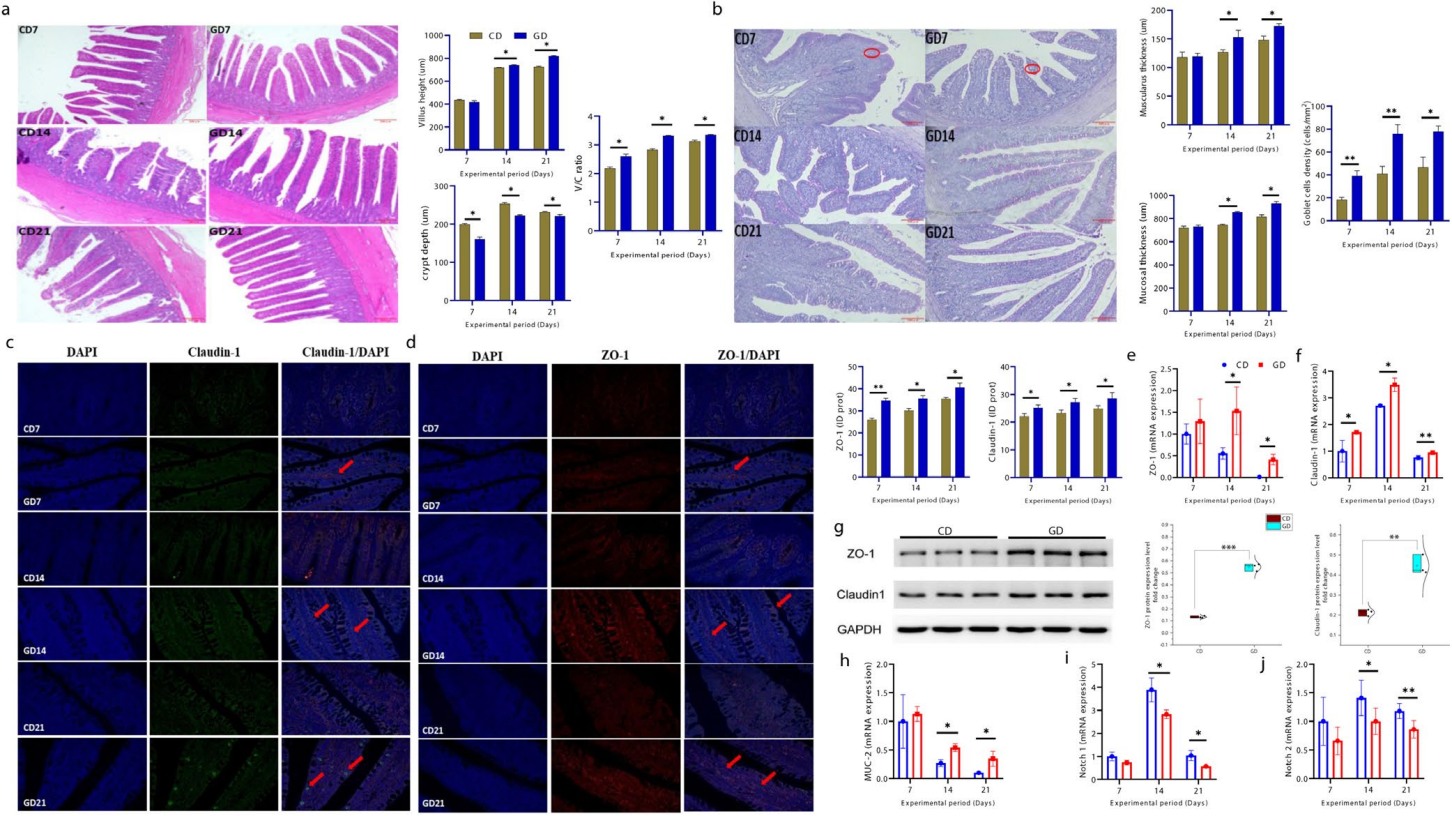
Perennial ryegrass preserves intestinal morphology and improves the intestinal integrity. (a) Representative images of H&E staining of ileum (*n* = 6), ileal morphological indices; villus height, crypt depth, villus height/crypt depth (V/C), respectively (b) representative images of cecal AB PAS staining (*n* = 6), muscularis thickness, mucosal thickness, goblet cell density, respectively (c, d) representative immunofluorescence expressions of Zonula Occludens‐1 (ZO‐1) and Claudin‐1 (ceca; *n* = 3), (e, f) relative gene expression levels of ZO‐1, claudin‐1 (ceca; *n* = 6), (g) representative western blotting images and quantification of proteins ZO‐1 and claudin‐1 (ceca; *n* = 3). GAPDH was used as internal control. (h–j) Relative gene expression levels of Mucin 2 (MUC2), neurogenic locus notch homolog protein 1 and 2 (Notch1 and Notch2) (ceca; *n* = 6). Data are expressed as mean ± SD. The asterisks symbol indicates significant differences *p < 0.05, **p < 0.01, ***P < 0.001.

Intestinal morphology was evaluated using hematoxylin and eosin (H&E) staining (Figure 2a). Ileal tissue villus height was significantly increased, whereas the crypt depth decreased in GD group (*p* < 0.05) as compared with the concentrate‐fed group. Villus height/crypt depth ratio (V/C) was also higher in GD group (*p* < 0.05) on Days 7, 15, and 21. Cecal morphology was further assessed by AB PAS staining (Figure 2b). The GD group demonstrated increased muscularis thickness, mucosal thickness, and goblet cell density relative to the CD group. The TJPs in cecal tissues were examined using immunofluorescence (IF). ZO‐1 and claudin‐1 protein levels were significantly higher in the GD groups (Figure 2c,d). Similar observations were found through mRNA expressions of ZO‐1 and claudin‐1 (Figure 2e,f). Protein expressions of ZO‐1 and claudin‐1 in ileum section were further verified by western blot images that showed higher expression in GD group (Figure 2g). These observations were in line with the IF and mRNA results. Additionally, cecal MUC2 gene expression was significantly upregulated in the GD groups (Figure 2h). In contrast, notch signaling pathway genes (Notch1 and Notch2) in ceca were highly expressed in CD group relative to GD (Figure 2i,j).

### 3.5 Perennial Ryegrass Preventing Systemic Inflammation

**FIGURE 3:**
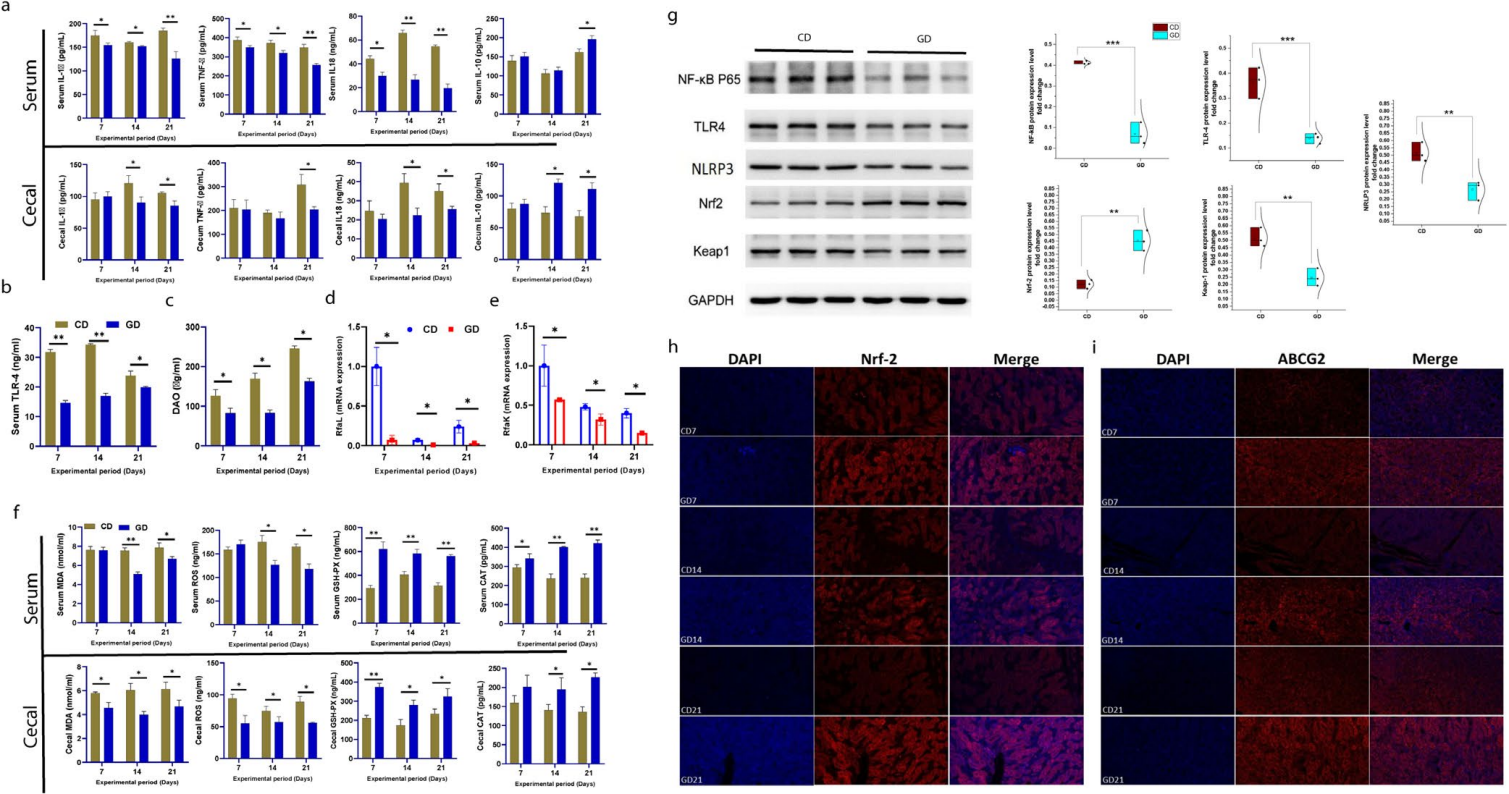
Perennial ryegrass alleviates oxidative stress. (a) Serum and cecal concentrations of inflammatory and anti‐inflammatory cytokines (IL‐1β, TNF‐α, IL‐18 and IL10), respectively (*n* = 6), (b) Serum concentration of TLR‐4 (*n* = 6), (c) Serum concentration of DAO (*n* = 6), (d, e) Gene expression of LPS producing genes from *rfa* cluster (*RfaK* and *RfaL*) (ceca; *n* = 6), (f) Serum and cecal concentrations of oxidants and antioxidants (MDA, ROS, GSH‐PX, and CAT), respectively (*n* = 6), (g) Representative western blotting images and quantification of proteins (NF‐κB, TLR4, NLRP3, Nrf2, and Keap1) in cecal tissues (*n* = 3). GAPDH was used as the internal control, (h, i) Representative immunofluorescence staining showing nuclear translocation Nrf2 and ABCG2 in kidney sections of wanpu geese (*n* = 3). Blue: DAPI‐stained nuclei; red: ABCG2 expression; merge: Combination of blue and red indicating nuclear translocation of ABCG2 at Days 7, 14, and 21, scale bar = 50 μum. Data are expressed as mean ± SD. The asterisks symbol indicates significant differences *p < 0.05, **p < 0.01, ***P < 0.001.

Pro‐inflammatory cytokines, including IL‐1β, TNF‐α, and IL‐18, were significantly reduced in both serum and cecal tissues in the GD group (*p* < 0.05) compared with the concentrate diet‐fed goslings (Figure 3a). In contrast, levels of the anti‐inflammatory cytokine IL‐10 were increased in the GD groups (Figure 3a). Gene expression analysis further supported these findings, as mRNA levels of IL‐1β, TNF‐α, and IL‐18 were higher in the CD group (Figure S3a–c). Whereas the gene expressions of IL‐10 and IL‐4 showed higher levels (*p* < 0.05) in the ryegrass‐fed group (Figure S3d,e). The transcriptional expressions were consistent with the results observed in serum and cecal tissues. Additionally, serum concentrations of toll‐like receptor 4 (TLR‐4) and diamine oxidase (DAO) were increased in the CD group (*p* < 0.05) as compared with GD (Figure 3b,c,g, Figure S3f). Genes involved in LPS biosynthesis within the rfa cluster (RfaK and RfaL) also increased in the CD group, as compared with the GD group (Figure 3d,e).

### 3.6 Perennial Ryegrass Enhanced Antioxidant Defense Through Nrf2‐ABCG2 Pathway

Ryegrass‐fed GD group showed lower serum and cecum level of oxidative mediator MDA and ROS as compared with CD (Figure 3f). higher level of GSH‐PX and CAT were observed in GD group (Figure 3f). transcriptional expressions for SOD and GSH‐PX showed significantly higher (*p* < 0.05) in GD group (Figure S3g,h). The relative mRNA and protein expressions of NF‐κB, MYD88, Keap1, and NLRP3 in cecal tissue were elevated in the CD group (*p* < 0.05) (Figure S3i–k, Figure 3g). GD group exhibited significantly higher ABCG2 expression at both mRNA and protein levels in cecal and renal tissues compared with the CD (Figure S2g,h, Figure 1f). The mRNA and protein expression levels of Nrf2 in cecal tissues were elevated in the GD group (Figure S3l, Figure 3g). Immunofluorescence analysis of kidney sections showed enhanced nuclear localization of Nrf2 (Figure 3h, Figure S4a) and increased ABCG2 expression (Figure 3I, Figure S4b) in the GD group, particularly on Days 14 and 21. These findings suggest a possible association between perennial ryegrass supplementation and modulation of the Nrf2‐ABCG2 signaling axis. Although concurrent upregulation of Nrf2 and ABCG2 was observed, the present study demonstrates correlation rather than direct causal activation of this pathway. Further mechanistic investigations would be required to establish a definitive regulatory relationship.

### 3.7 Perennial Ryegrass Modulated Gut Microbial Diversity

**FIGURE 4:**
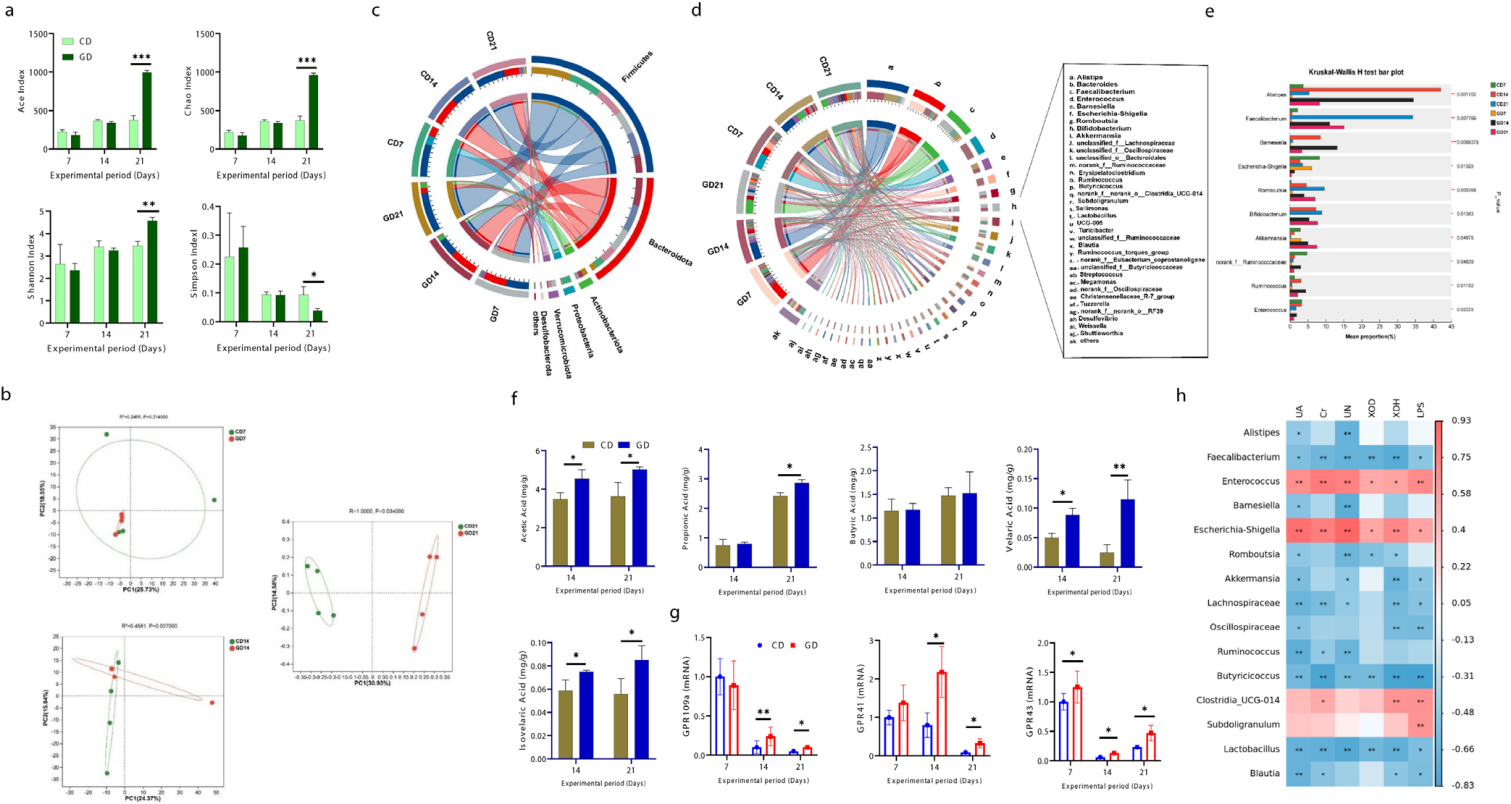
Inclusion of perennial ryegrass improved gut microflora. (a) Alpha diversity indexes; Ace, Chao, Shannon, and Simpson indexes, (b) Beta diversity; principal coordinates analysis (PCoA) between the experimental groups at Days 7, 14, and 21, (c) Circos diagram at phylum level, (d) Circos diagram at genus level, (e) Bacterial abundance at genus level, (f) Shot‐chain fatty acids concentrations in ceca (*n* = 6), (g) Gene expressions of shot‐chain fatty acids receptors in ceca (*n* = 6), (h) Cross talk between gut microbes and HUA indices. *P < 0.05, **P < 0.01, ***P < 0.001.

Microbial composition is mainly modulated by dietary composition in both animals and humans. Microbial community structure was characterized by 16S rRNA gene sequencing. Alpha diversity was evaluated using richness indices (Ace and Chao) and diversity indices (Shannon and Simpson) on Days 7, 14, and 21 (Figure 4a). GD group exhibited significantly higher alpha diversity (*p* < 0.05), particularly on Day 21. PCoA based beta diversity analysis revealed distinct clustering patterns among treatments, with significant microbial separation observed in the GD groups on Days 14 and 21 (Figure 4b). At the phylum level, Firmicutes and Bacteroidetes were the dominant taxa in both dietary groups, as illustrated by the Circos (Figure 4c). Notably, Bacteroidetes showed higher relative abundance in the ryegrass‐fed goslings. At the genera level, Circos diagram representing the bacterial diversity and abundance in CD and GD groups (Figure 4d). As described by Kruskal‐Wallis H test, *Alisteps, Faecalibacterium, Barnesiella, Akkermansia*, and *Ruminococcus* were abundantly present in GD group compared to CD (Figure 4e), while the abundance level of *Enterococcus* and *Escherichia‐Shigella* were higher in CD group (*p* < 0.05). Short‐chain fatty acids levels were significantly increased in the GD group, while butyric acid was not influenced significantly (Figure 4f). The cecal gene expressions of short‐chain fatty acid receptors were also measured and showed an increasing trend in the GD group (Figure 4g).

### 3.8 Perennial Ryegrass‐Based Diet Modulated Microbial Homeostasis

Spearman's correlation analysis revealed that the GD group exhibited greater microbial evenness and distinct correlations with the CD group on Days 7, 14, and 21 (Figure S5a). Interactive Pearson correlation analysis showed cross‐talk between gut microbes and HUA indices (Figure 4k). The analysis described that CD‐enriched genera, including *Enterococcus, Escherichia‐Shigella*, *Clostridia UCG‐014*, and *Subdoligranulum*, were positively correlated with HUA indicators. In contrast, GD‐enriched taxa such as *Faecalibacterium*, *Lachnospiraceae*, *Butyricicoccus*, *Lactobacillus*, and *Akkermansia* were significantly negatively correlated with HUA indicators (*p* < 0.05). *Alistipes*, *Ruminococcus*, and *Blautia* also showed negative associations. The results suggest that perennial ryegrass supplementation may increase beneficial microbes while reducing opportunistic pathogens, thereby potentially enhancing disease resistance in geese.

### 3.9 Differentially Expressed Metabolites

**FIGURE 5:**
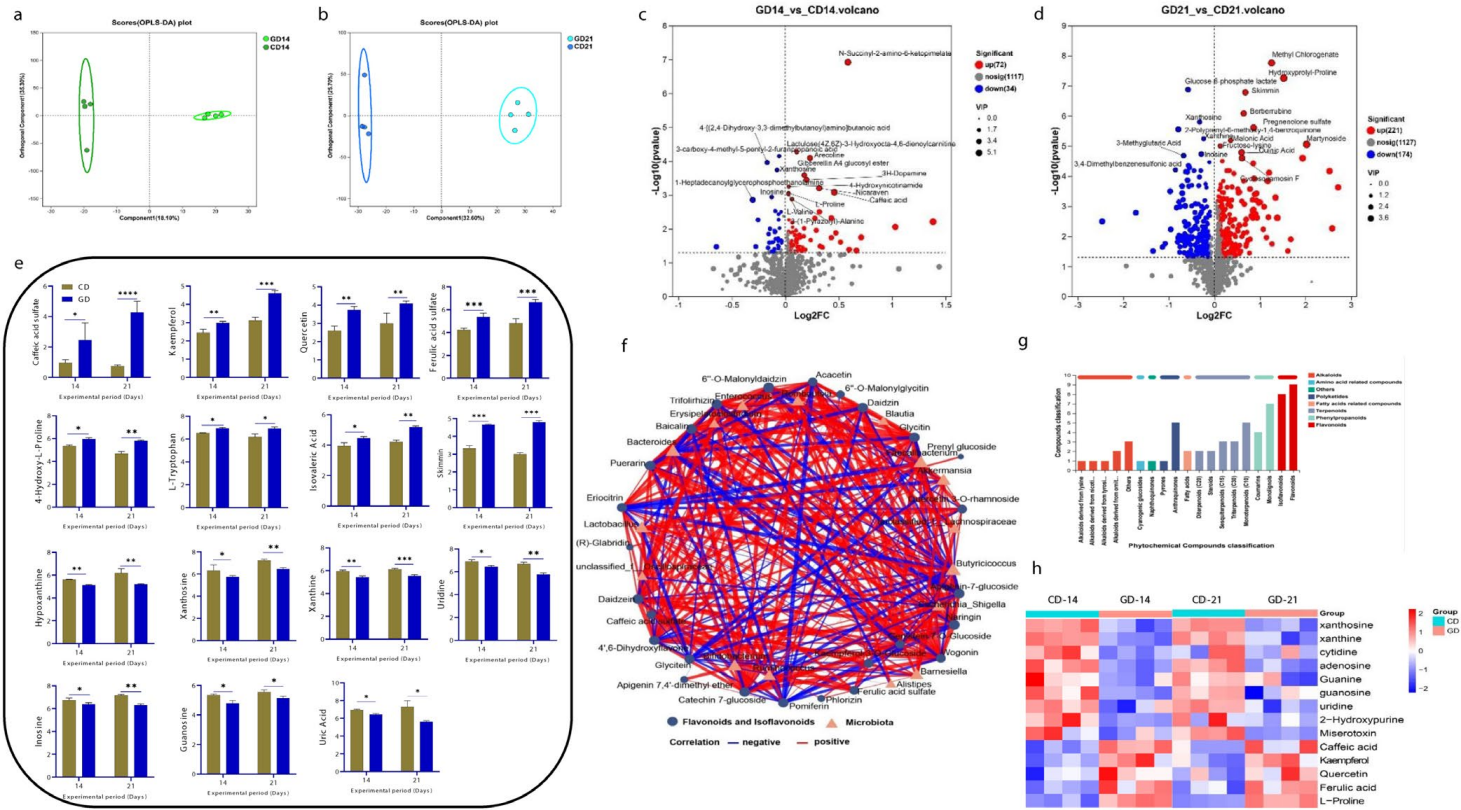
Perennial ryegrass modulated the differentially expressed metabolites and reduced the HUA related metabolites. (a, b) OPLS‐DA score plots at Days 14 and 21, (c, d) Volcano diagram represents differentially expressed metabolites at Days 14 and 21, (e) Differentially expressed metabolites at Days 14 and 21, (f) Network correlation diagram between microbiota, and flavonoids and isoflavonoids, (g) Phytochemical compounds classification and enrichment analysis at Day 21, (h) Heat map analysis of LC–MS metabolites between experimental groups at Days 14 and 21. *P < 0.05, **P < 0.01, ***P < 0.001.

In serum metabolomics analysis, a clear separation among groups was showed by the OPLS‐DA score plot with high reliability using the permutation test (R^2^Y (cum) = 0.994, Q^2^ (cum) = 0.921) for Day 14 and (R^2^Y (cum) = 0.996, Q^2^ (cum) = 0.83) for Day 21 (Figure 5a,b). Volcano plot analysis illustrated the differential metabolites, at Day 14, (total = 1223, up = 72 and down = 34) and at Day 21 (total = 1522, up = 221 and down = 174) (Figure 5c,d). The antioxidant metabolites (Caffiec acid, kaempferol, and Quercetin) were up‐regulated and the metabolites related to UA metabolism were down‐regulated. The abundance of antioxidant metabolites and HUA related metabolites were analyzed in both groups at Days 14 and 21 (Figure 5e). The metabolites (caffiec acid, kaempferol, quercetin, and ferulic acid) showed higher abundance in GD group. The abundance of l‐proline, l‐tryptophan, isovaleric acid and skimming also higher in GD group. While compared with GD, the abundance of HUA related metabolites (hypoxanthine, xanthosine, xanthine, uridine, inosine, guanosine, and UA) was higher in CD group. Network correlation analysis described the relationship between gut microbial communities and polyphenolic metabolites (Figure 5f). The opportunistic bacteria, mainly *Enterococcus* and Escherichia‐Shigella showed the negative associations, while the probiotic genra mainly *Akkermansia*, *Ruminococcus, Lactobacillus*, and *Romboutsia* showed positive correlations with bioactive polyphenols, including caffeic acid, quercetin, kaempferol, and ferulic acid.

### 3.10 Perennial Ryegrass Enhanced the Production of Antioxidant Metabolites

Perennial ryegrass significantly increased the abundance of polyphenolic metabolites, particularly flavonoids, isoflavonoids, monolignols, and monoterpenoids, on Days 14 and 21 (Figure S5b, Figure 5g). Heat map correlation analysis compared the metabolite profiles associated with uric acid metabolism among treatments (Figure 5h). The concentrations of UA associated metabolites (xanthosine, xanthine and hydroxypurine) were higher in CD group, whereas the concentration of different antioxidant metabolites, that is, caffeic acid, quercetin, kaempferol, and ferulic acid was increased in GD group. At Days 14 and 21, amino acids and lipid metabolism pathways were primarily influenced by ryegrass inclusion in Pathways enrichment analysis (Figure S5c,d). pathways related to purine and pyrimidine metabolism, thermogenesis, and inflammatory mediators were significantly downregulated in GD group. Metabolite Correlation analysis demonstrated negative associations between phytochemical metabolites and HUA‐associated metabolites (hypoxanthine, xanthine, and inosine) (Figure S6a,b). These findings may indicate the possibility that perennial ryegrass supplementation may modulate purine‐related metabolites and promote the synthesis of bioactive compounds associated with improved metabolic health in Wanpu geese.

## 4 Discussion

**FIGURE 6:**
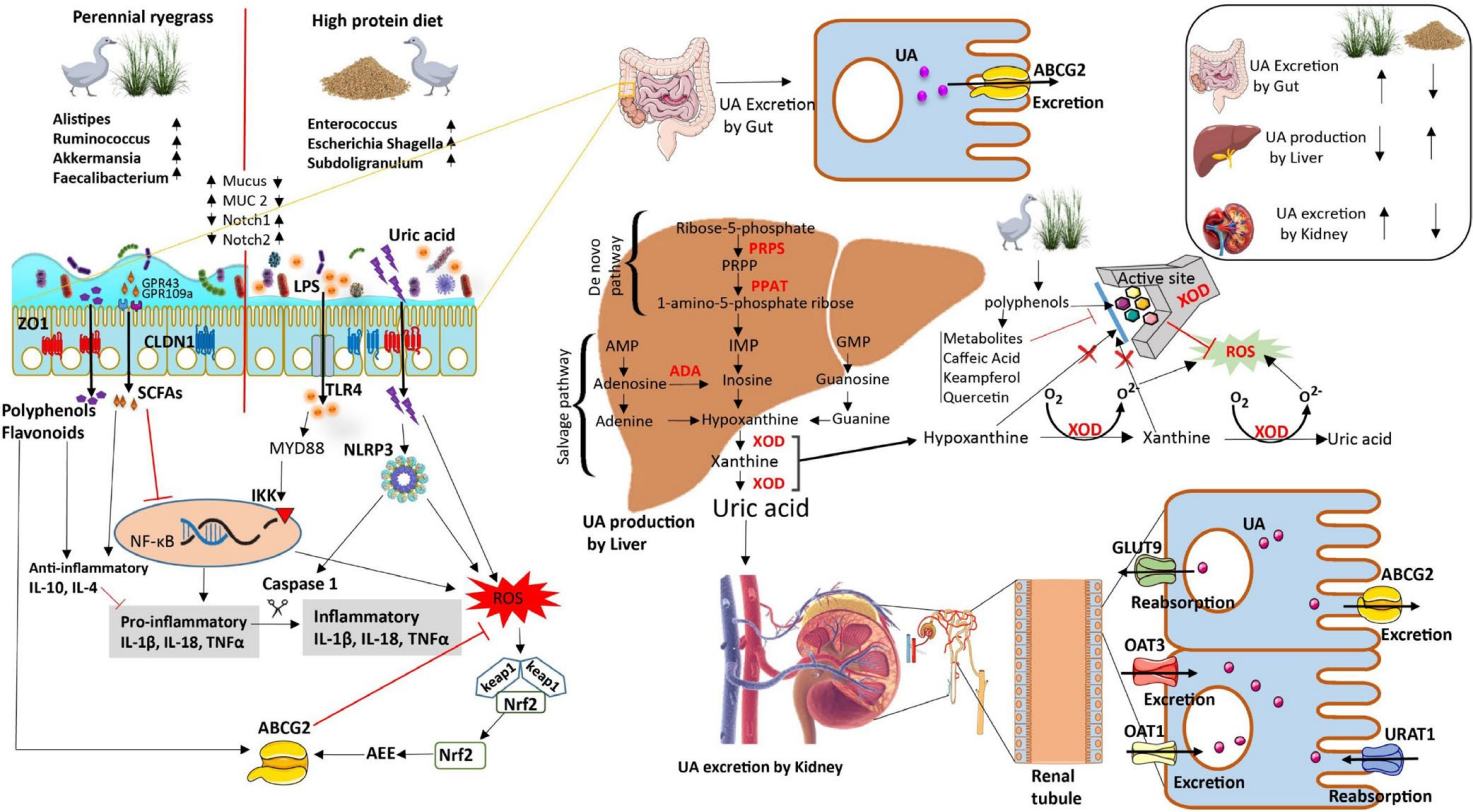
Schematic of perennial ryegrass‐mediated protection against hyperuricemic‐oxidative stress via activating antioxidant Nrf2‐ABCG2 pathway. The dietary inclusion of perennial ryegrass enhances intestinal health by promoting beneficial SCFA‐producing bacteria, increasing mucus production through upregulation of MUC2 and goblet cells, and strengthening gut barrier integrity (upregulating ZO‐1 and claudin1) while suppressing Notch1/2 expression. It alleviates HUA by downregulating hepatic UA‐synthesizing enzymes (XOD and XDH), meanwhile reduced superoxides production. It upregulating UA‐excreting transporters (ABCG2, OAT1, OAT3) in the intestine and kidney. Additionally, perennial ryegrass enhanced antioxidant metabolite production and mitigated ROS‐mediated oxidative stress by activating the Nrf2‐ABCG2 antioxidant signaling pathway in goslings.

Hyperuricemia (HUA) is a progressive metabolic risk factor associated with gout, chronic kidney disease, type 2 diabetes, and cardiovascular disorders worldwide. One of the major constraints in elucidating uric acid (UA) metabolism is the limited availability of suitable animal models, as most mammals possess uricase activity (Fu et al. 2024). In contrast, birds, including geese, lack uricase and therefore represent a physiologically relevant model for investigating UA metabolism and HUA‐associated pathologies. Rapid growth rates and high‐protein concentrate diets commonly used in goose production can exacerbate purine metabolism, increasing susceptibility to HUA and avian gout (Wang et al. 2025). These conditions not only compromise animal welfare and productivity but also result in substantial economic losses to the goose industry (Xi et al. 2019). Given the increasing global demand for goose meat, particularly in China, the development of sustainable nutritional strategies to mitigate HUA is of both scientific and economic importance. Allopurinol has long been used as a first‐line pharmacological therapy for hyperuricemia; however, its application is associated with renal adverse effects and, in severe cases, fatal hypersensitivity reactions (Alghamdi et al. 2020). Moreover, its use in food‐producing animals is restricted due to food safety and regulatory concerns. Consequently, recent studies have increasingly focused on dietary modulation and plant‐based interventions to elucidate HUA pathophysiology and develop safer preventive strategies in poultry (Xi et al. 2019; Wang et al. 2021). For example, chicory supplementation in hyperuricemic quail has been shown to exert antioxidant, anti‐inflammatory, and urate‐lowering effects (Bian et al. 2020). In the present study, Wanpu goslings were used to evaluate the high‐fiber and antioxidant potential of perennial ryegrass as a dietary component for mitigating HUA. Our findings suggested that dietary inclusion of perennial ryegrass may be associated with improvements in intestinal health, modulation of gut microbiota composition, UA metabolism via the gut‐liver‐kidney axis, and cellular redox homeostasis. Collectively, these coordinated changes suggest that perennial ryegrass supplementation may contribute to modulate UA metabolism in geese (Figure 6).

However, it is important to emphasize that the present findings primarily reflect coordinated associations rather than direct causal relationships. The proposed gut microbiota–antioxidant pathway should therefore be interpreted as a conceptual mechanistic framework supported by multi‐omics convergence, rather than definitive proof of causality. Although beneficial shifts in microbial composition, polyphenolic metabolite enrichment, enhanced antioxidant responses, and improvements in UA metabolism occurred concomitantly, direct cause–effect relationships cannot be established based on the current experimental design. Future studies employing targeted approaches, such as microbiota depletion, fecal microbiota transplantation, gnotobiotic models, or controlled microbial and metabolite interventions will be required to distinguish microbiota‐dependent from microbiota‐independent mechanisms underlying the protective effects of perennial ryegrass.

Growth performance analysis revealed that goslings fed the concentrate diet exhibited higher average daily gain (ADG) than those receiving ryegrass‐supplemented diets, likely due to the greater caloric density of the concentrate feed. In contrast, goslings in the perennial ryegrass groups displayed an improved lipid profile, which may be attributed to the high fiber content and antioxidant bioactive compounds present in ryegrass. Mortality was observed in the control group within the normal range and may be associated with early‐adaptation stress or natural biological variation rather than treatment effects. These observations are consistent with previous studies demonstrating that pasture‐fed geese exhibit reduced fat deposition, improved lipid metabolism, and favorable carcass characteristics with potential economic benefits (Guan et al. 2013; Song et al. 2017; Ali et al. 2022; Şişman and Tilki 2024).

The disruption to blood chemistry induced by high blood UA is one of the most identifiable factors in HUA and avian gout (Desai et al. 2017). A limitation of the present study is the absence of a well‐defined hyperuricemia disease model or positive‐control group. Although the goslings fed the concentrate diet exhibited elevated serum UA and creatinine (Cr) levels, a pathological HUA state was not experimentally induced. Therefore, the findings should be interpreted as evidence of metabolic regulation rather than definitive prevention or treatment of established HUA. Whereas the ryegrass‐fed group significantly modulated these markers. These findings are consistent with previous reports showing increased UA, Cr, and blood urea nitrogen levels in birds with HUA and visceral gout (Guo et al. 2005; Wang et al. 2011; Xi et al. 2022; Fu et al. 2024). The reduced UA levels observed in the ryegrass‐fed group were associated with lower xanthine oxidase (XOD) expression, which is in line with the reported inhibitory effects of polyphenols such as quercetin, caffeic acid, and kaempferol on XOD activity (Saw et al. 2014; Gessner et al. 2017). These compounds are known to interact with XOD active sites, thereby limiting the conversion of hypoxanthine and xanthine into UA (Dong et al. 2016). Excessive XOD activity has been implicated in renal dysfunction and oxidative stress, both of which contribute to the development of HUA and avian gout (Hainer et al. 2014; Lin et al. 2016; Xi et al. 2019).

Dietary inclusion of perennial ryegrass may also be associated with enhanced expression of UA excretion transporters (ABCG2, OAT1, and OAT3) and reduced expression of reabsorption transporters (URAT1 and GLUT9), suggesting a shift toward increased UA clearance. These observations are consistent with previous studies reporting that plant‐derived polyphenols modulate renal and intestinal UA transport, thereby facilitating UA elimination (Perez‐Ruiz et al. 2015; Chen et al. 2015; Lee et al. 2017; Bian et al. 2020). Furthermore, ABCG2 is a key UA transporter involved in intestinal and renal excretion and has also been implicated in cellular protection against oxidative stress (Nie et al. 2018). Keap1‐Nrf2 signaling plays a central role in cellular redox regulation, with Nrf2 known to regulate antioxidant enzymes and potentially influence ABCG2 expression via antioxidant response elements (Singh et al. 2010). In the present study, perennial ryegrass supplementation was associated with enhanced Nrf2‐related antioxidant responses and increased ABCG2 expression, accompanied by elevated antioxidant enzyme activity and reduced oxidative stress markers. These findings suggest a potential involvement of Nrf2‐ABCG2‐related antioxidant mechanisms, although direct mechanistic activation cannot be confirmed without further pathway‐specific studies.

The gut is one of the largest endocrine organs, harboring thousands of microbial communities in the body (Masetti 2022). These microbial communities can be altered by dietary interventions in a rapid and diet‐specific manner (David et al. 2014). In this study, perennial ryegrass intervention in the GD group was associated with the enrichment of beneficial microbial taxa, including *Alistipes, Ruminococcus, Bacteroides, Akkermansia, Lactobacillus*, and *Butyricicoccus*, alongside reduced abundance of opportunistic gram‐negative bacteria such as *Enterococcus* and Escherichia‐Shigella. These microbial shifts coincided with improvements in short‐chain fatty acid production, intestinal morphology, and tight junction protein expression, suggesting enhanced gut barrier function. Similar microbiota‐modulating effects of perennial ryegrass and dietary fiber have been reported previously (David et al. 2014; Zheng et al. 2021; Ali et al. 2022). Nevertheless, these observations should be interpreted as associative, and further studies are required to determine whether microbial alterations directly mediate the observed metabolic and antioxidant benefits. The inflammatory and anti‐inflammatory cytokines measured from serum and ceca reflect integrated systemic and intestinal inflammatory status rather than tissue‐specific sources. The concurrent reduction of pro‐inflammatory cytokines at both levels, together with improvements in intestinal barrier integrity and oxidative stress markers, suggests a link between intestinal and systemic inflammatory modulation. However, these findings do not establish the gut as the primary source of systemic inflammation, as serum cytokines may originate from multiple tissues and cecal cytokines may reflect both host‐derived and intestinal immune signaling. Thus, the observed inflammatory changes are best interpreted as reflecting bidirectional gut‐systemic immune interactions rather than a unidirectional gut‐derived process.

Additionally, Metabolomics approaches were applied to elucidate blood metabolic changes established due to experimental diets in young goslings. Findings of present study revealed that the metabolites mainly related to the amino acid metabolism and lipid metabolism were expressed differently. Arginine, proline and tryptophan metabolisms were up‐regulated in GD group, meanwhile cholesterol metabolism, purine, histidine, thermogenesis and reactive oxygen species metabolism were down‐regulated. By intervention of perennial ryegrass the production of flavonoids, isoflavonoids and terpenoids compound enhanced in GD group and augmented the antioxidant defense mechanism. The results of current study were consistent with previous studies (Wu and You 2022; Wu et al. 2022; Fu et al. 2024). Bian et al. (2020) showed that by supplementation of chicory in hyperuricemic quail diets increased the production of Flavonoid and terpenoids (Bian et al. 2020). Fu et al. (2024) described that up‐regulation of proline reduced the HUA by inhibiting the conversion of adenosine to inosine or UA by suppressing the ADA gene (Fu et al. 2024). Using glycine and tryptophan as a treatment therapy in Mild HUA patients, reduced the risk of monosodium urate crystal deposition and increased the uric acid excretion (Oshima et al. 2019). These results suggested that perennial ryegrass inclusion in diet could significantly enhance gosling health by fostering a healthy gut microbiome and their mediated metabolites. We recommend perennial ryegrass as diet portion which may enhance the production of antioxidant metabolites, modulate gut microbial diversity, UA metabolism and redox homeostasis for improving gosling's health.

## 5 Conclusion

The present study demonstrates that dietary composition influences uric acid metabolism and metabolic health in young goslings. Intervention of perennial ryegrass with concentrate diet (30:70) as a plant‐based high fiber could be a potential nutritional strategy to modulate UA metabolism and antioxidant potential in young goslings. Although the mechanistic basis is still unclear, it likely enhanced the probiotic microbial communities, which produced short‐chain fatty acids and other antioxidant metabolites. These metabolites may reduce the activity of UA‐producing enzymes and up‐regulate the UA‐excreting transporters' activity. Inclusion of perennial ryegrass improved intestinal morphology and mucosal integrity. It mainly influenced amino acid and lipid metabolisms, increased the production of antioxidant enzymes. Although a pathological hyperuricemia model was not established in this study, the findings indicate that moderate inclusion of perennial ryegrass (30%) in concentrate‐based diets beneficially modulates UA metabolism, antioxidant defenses, and intestinal health.

## Author Contributions

**Muhammad Arslan Asif:** writing‐original draft, formal analysis, investigation, and methodology. **Zeshan Zulfiqar:** data curation and conceptualization. **Bahar E. Mustafa:** review and editing. **Saira Saif** and **Sun Hao:** data curation. **Zhichang Wang** and **Yalei Cui:** methodology, and review. **Yinghua Shi** and **Liu Boshuai:** resources, supervision, project administration, funding acquisition.

## Funding

This work was supported by China Agriculture Research System of MOF and MARA (No. CARS‐34) and Science and Technology Innovation Leading Talent in Central Plains (No. 244200510010).

## Ethics Statement

Protocols used for animal collection were approved by the Henan Agriculture University Animal Care Committee.

## Conflicts of Interest

The authors declare no conflicts of interest.

## Supporting information


**Data S1:** fsn371760‐sup‐0001‐Supinfo.docx.

## Data Availability

The data that support the findings of this study are available from the corresponding author upon reasonable request.
