# Supplementary material for: Nutritional Intervention With Perennial Ryegrass Modulates UA Metabolism in Goslings via Gut Microbiota–Antioxidant Pathway Modulation
Source: Food Sci Nutr. 2026 Apr 17;14(4):e71760. doi: 10.1002/fsn3.71760 (PMC13088338; doi:10.1002/fsn3.71760)
Supplement: Supplementary file 1 — Data S1: fsn371760‐sup‐0001‐Supinfo.docx. [file FSN3-14-e71760-s001.docx]

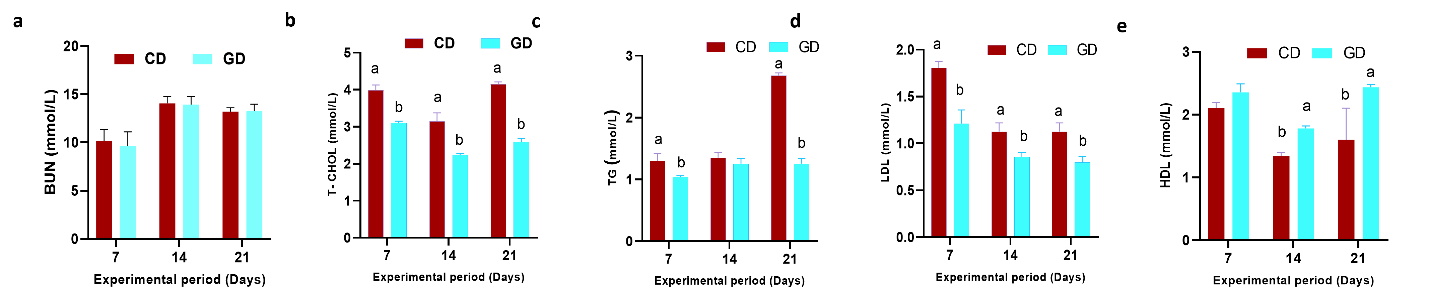


**Figure S1. Effect of perennial ryegrass on lipid profile indices and UA producing enzymes and UA excreting transporters gene expressions. a** blood urea nitrogen (BUN), **b** total cholesterol (T-CHOL), **c** triglycerides (TG), **d** low density lipoprotein (LDL), **e** high density lipoprotein (HDL).

**
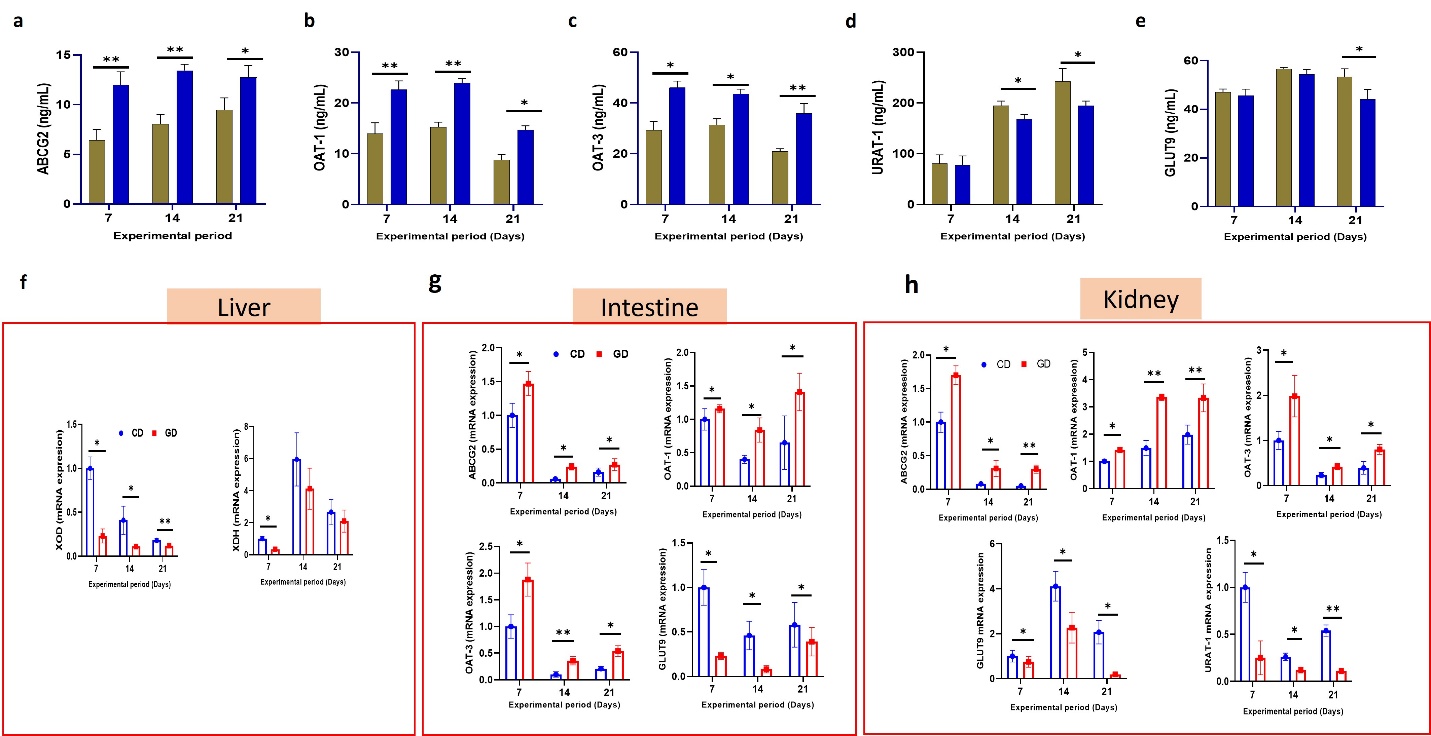
**

**Figure S2. Effect of perennial ryegrass on lipid profile indices and UA producing enzymes and UA excreting transporters gene expressions. a-e** Serum levels of ATP-binding cassette subfamily G member 2 (ABCG2), organic anion transporter 1 and 3 (OAT1, OAT3), urate transporter 1 (URAT1), and glucose transporter 9 (GLUT9) respectively. **f** gene expressions of UA producing enzymes (XOD and XDH) in liver, **g** gene expressions of UA excreting transporters (ABCG2, OAT1 and OAT3) and reabsorbent (GLUT9) in intestine, **h** gene expressions of UA excreting transporters (ABCG2, OAT1 and OAT3) and reabsorbents (GLUT9) in kidney


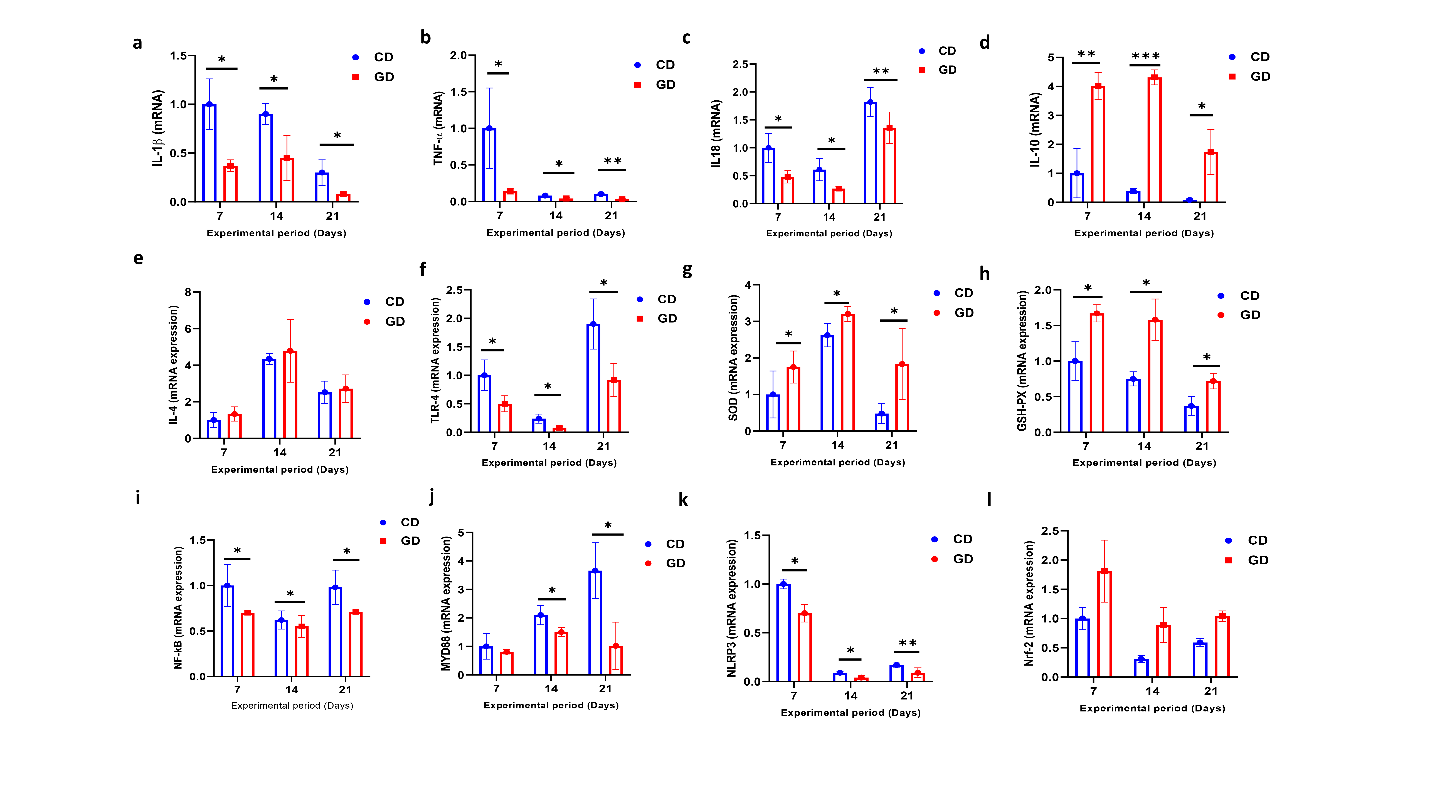


**Figure S3. Effect of perennial ryegrass on inflammatory and anti-inflammatory cytokines. a** gene expressions of IL-1β, **b** gene expressions of TNF-α, **c** gene expressions of IL-18, **d** gene expressions of IL10, **e** gene expressions of IL-4, **f** gene expressions of TLR-4, **g** gene expressions of SOD, **h** gene expressions of GSH-PX, **i** gene expressions of NF-κB, **j** gene expressions of MTD88, **k** gene expressions of NLRP3, **l** gene expressions of Nrf-2


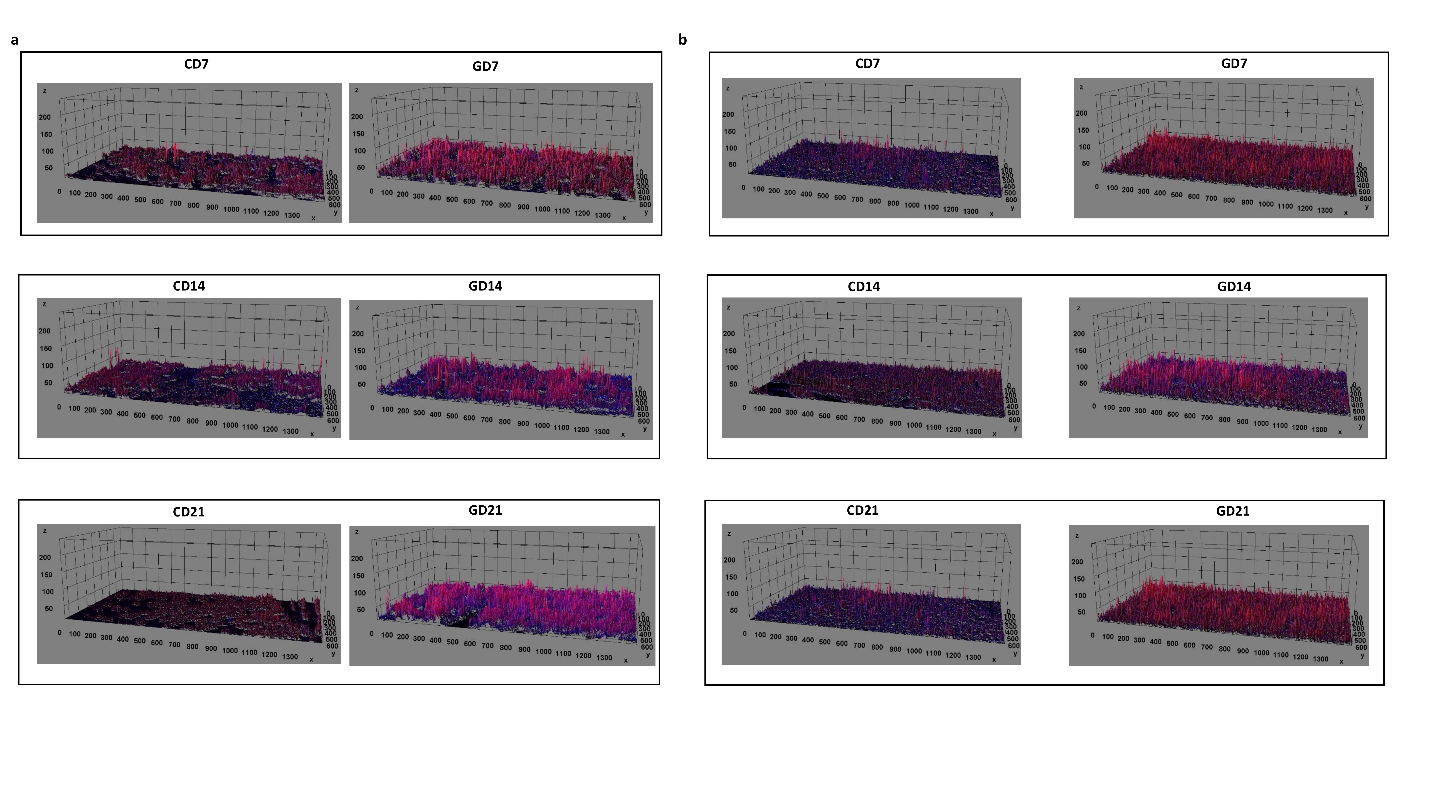


**Figure S4. a-b** D surface plots of immunofluorescence intensities was analyzed using Image J software analysis.


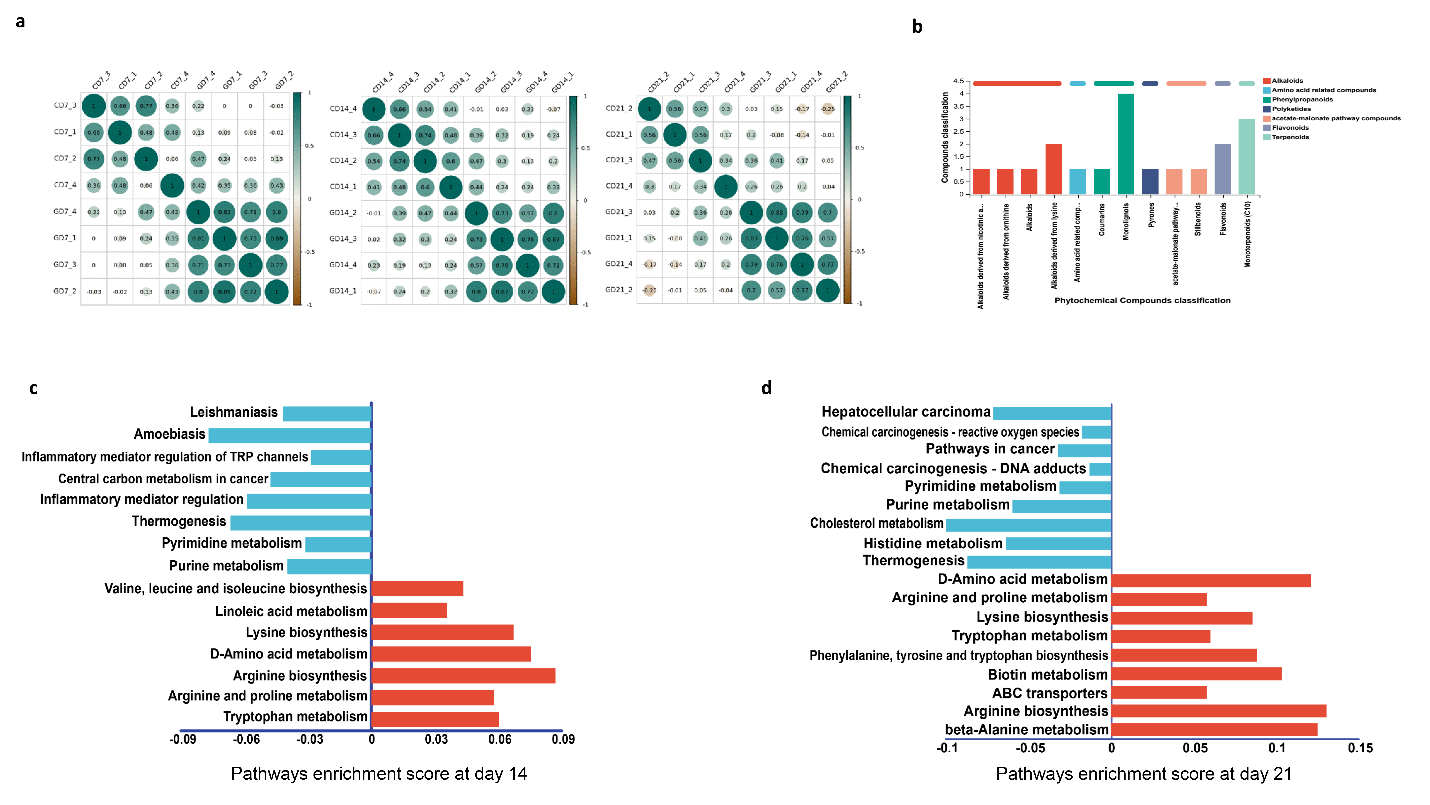


**Figure S5. Inclusion of perennial ryegrass altered the micro flora and modulated the metabolic pathways. a** Spearman correlation analysis at genus level between experimental groups at days 7, 14 and 21**,** **b** Phytochemical compounds estimation at day14, **c** Pathways enrichment score at day 14, **d** Pathways enrichment score at day 21


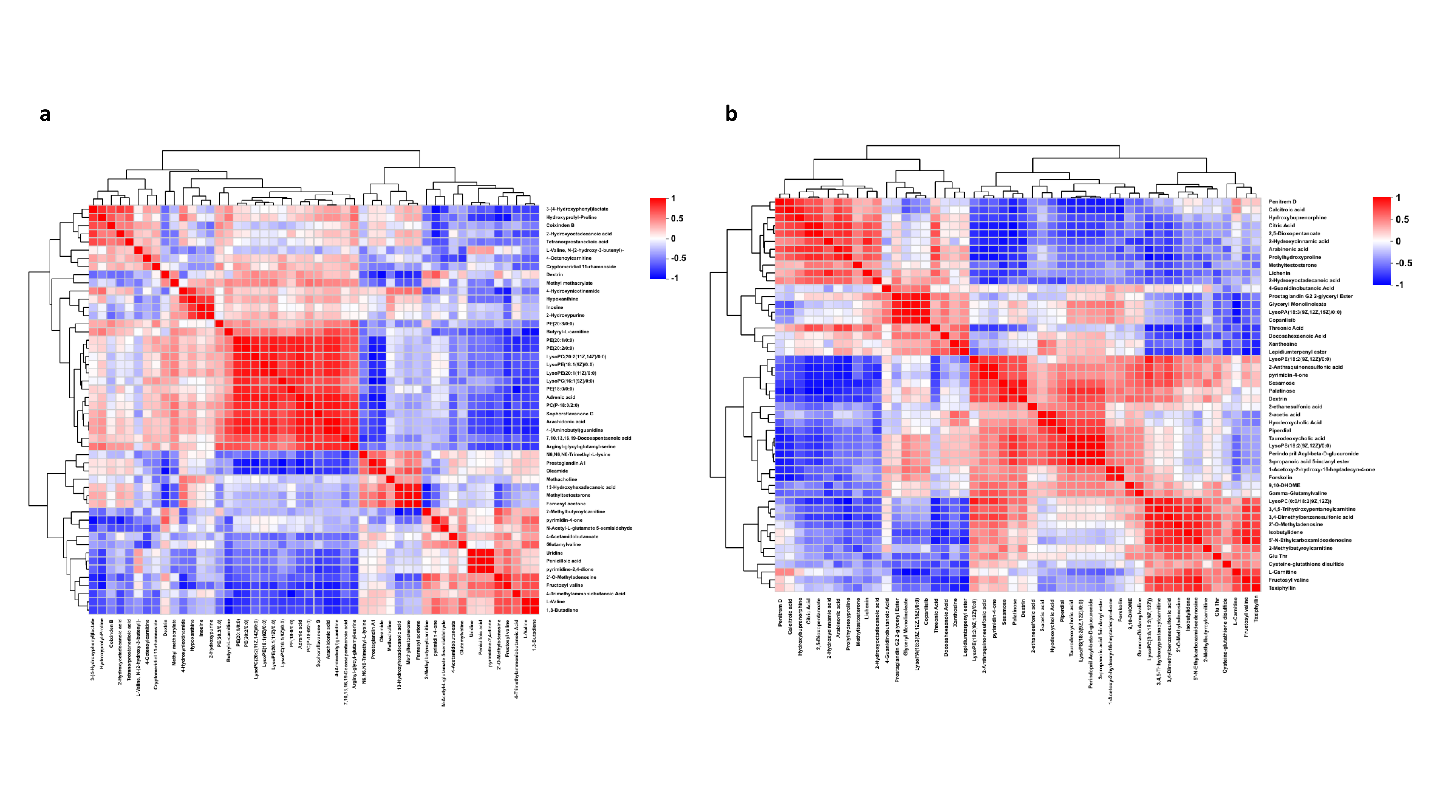


**Figure S6. Perennial ryegrass enhanced the production of beneficial metabolites. (a-b)** Correlation analysis of differential metabolites is conducive to further understanding the mutual regulation relationship between metabolites at days 14 and 21,

**Table S1.** Commercial feed formulation used in the study

| **Ingredients %** | **0-21 day** |
| --- | --- |
| Corn | 57.8 |
| Wheat Bran | 5.0 |
| Soybean Meal (43.5%) | 30 |
| Rice Husk | 1.3 |
| Sunflower meal | 2.00 |
| Limestone | 1.1 |
| Dicalcium phosphate (DCP) | 1.4 |
| Lysine | 0.04 |
| DL methionine (DLM) | 0.1 |
| Premix | 1 |
| Salt | 0.3 |
| ME (MJ/Kg) | 11.4 |
| Crude Protein | 19.57 |
| Crude Fiber | 4.50 |
| Ash | 12.89 |
| Calcium | 0.83 |
| Total Phosphorus | 0.65 |
| Lysine | 0.92 |
| Methionine | 0.37 |

Premix provided per kilogram of diet: vitamin A, 2000 IU; vitamin D3, 45000U; vitamin E, 300IU; vitamin K3, 20 mg; vitamin B1, 10 mg; vitamin B2, 120 mg; vitamin B6, 20 mg; nicotinic acid, 600 mg; pantothenic acid, 180 mg; folic acid, 10 mg; choline, 7 g; Fe, 1.2 g; Cu, 0.2 g; Mn, 1.9 g; I, 10 mg; Se, 6 mg.

**Table S2.** Nutrients composition of perennial ryegrass

| **Nutrients** | **Specifications** |
| --- | --- |
| Dry matter (DM) | 20% |
| Crude protein (DM) | 17.6% |
| Metabolizable energy (MJ/kg, DM) | 9.5 |
| Ether extract (DM) | 3.3% |
| Ash (DM) | 8.7% |
| Crude fiber (DM) | 26.5 |
| Neutral detergent fiber (NDF) DM | 36 |
| Acid detergent fiber (ADF) DM | 22 |
| Calcium (g/kg, DM) | 0.9 |
| Phosphorus (g/kg, DM) | 0.47 |

**Table S3.** Primer sequences used for quantitative real-time PCR

| **Gene name** | **Forward primers (5′ to 3′)** | **Reverse primers (5′ to 3′)** |
| --- | --- | --- |
| XOD | ACTGTCGAAGGCATAGGA | GCTGGAACTCGGAAGAAT |
| XDH | CGAGCTCAGCACACAGGTAA | TAGACCCTCACAGACCAGGG |
| OAT1 | CTGCATCTTCCTGTACACTG | CGTAGATGAAGAGAGGCATG |
| OAT3 | CTCTTGAGGACGATCCTGGC | CGGGCCCACACGTTACTTAT |
| ABCG2 | ACCCAGTTCACTCGTGGG | TCCAGTCCAGTGGTTGGCTC |
| GLUT9 | CAACTGGCTCTCCAACTTCG | CTTGGCTGATCTCATTGAGTGT |
| URAT1 | GGCTTCACCTTCTACGGCCT | AGCAGCAGGGTGCCGATCTT |
| ZO-1 | ACGCTGGTGAAATCAAGGAAGAA | AGGGACATTCAACAGCGTGGC |
| Claudin-1 | CGATACTCCTGGGCCTTGTC | ACCAATGCTGATAAACCTGCAA |
| MUC 2 | GCCTTTCACTCAGCAGCTTT | CCATGACTCACCTGGCTGTA |
| GPR43 | TAGAACGCTACCTGGGAGTG | ACCAGAGCAGCGATCACTC |
| GPR41 | TTGACGCCAAGAAGCGGTAT | CGAGACATGCATTCAGCGTG |
| GPR109a | AATGGTGTCCCGCTACAAGG | GTCTCCACACACGCAAAACC |
| Notch1 | GTGAAATCGATGCGGACTGC | ATGAAGTCGGAGATGACGGC |
| Notch2 | CGCATCCGTGCTTGAACAAA | AGGGAGACCTGCTGCATAGA |
| RfaL | TGTTCACTTCTCCTAGACAGCA | CTTGTCCTTTCAGAAGTGGCA |
| RfaK | CCTCTTCTGCTGGCACTACT | TTCCCATCCCCAGCAGAAAT |
| SOD | CGCTATCAAGTTCAACGGCG | GCACCCTCAGCACTCATCTT |
| GSH-PX | GGACACCAGGAGAATGGCAA | GTAAAGAGCGGGTGAGCCTT |
| IL-1B | CACATCACAACCCACAGCAA | CTGCCCCTTCCGTCTTCTTA |
| IL-18 | CTGCCTCTACTTTGCTGACGA | ACCACAAGCACCTGGCTATT |
| TNF-α | GGTCCACAACGAGTTCATCC | AGGAGGAGGAGGAGATGGAG |
| IL-10 | CATCAAGAACAGCGAGCACC | GCACCCACCTTTTCAAACGT |
| IL-4 | TGACAGGGTATTGGTCCACC | ACGGAAGAAGCAGAAGGTGA |
| NF-κB | GGCAGAGATGGTGGAAGACT | GTTTGCCATCACCACCATGT |
| TLR-4 | AGGGCTACAGGTCAACAGAC | GACGTTCACCAGCCGAATAC |
| MYD88 | AGCGTGCCAAAGACTTCAGA | ACACGTTCCTGGCAAGACAT |
| NLRP3 | CTCTGCTCAGCACCACGAGA | TCCACATGCCGAGGATGGTC |
| Nrf-2 | CGCCTTGAAGCTCATCTCAC | CCTCTCCTGCGTATATCCCG |

**Table S4.** Antibodies used in this study for western blot

| **Antibodies** | **KDa** | **Source** | **Catalog No.** |
| --- | --- | --- | --- |
| GAPDH | 36 | Proteintech | 60004-1-Ig |
| OAT-1 | 55 | Bioss | bs-0606R |
| ABCG2 | 68-72 | 万类生物 | WL03192 |
| URAT1 | 60 | Proteintech | 14937-1-AP |
| GLUT9 | 59 | Proteintech | 26486-1-AP |
| OAT-3 | 65 | Bioss | bs-0609R |
| XOD | 146 | Proteintech | 55156-1-AP |
| ZO-1 | 195 | Affinity | AF5145 |
| Claudin1 | 20-23 | proteintech | 13050-1-AP |
| NF-κB P65 | 65 | proteintech | 10745-1-AP |
| NLRP3 | 85-110 | Abclonal | A5258 |
| Nrf2 | 100 | Abclonal | A1244 |
| Keap1 | 70 | Bioss | bs-3648R |
